# From Culture-Medium-Based Models to Applications to Food: Predicting the Growth of *B. cereus* in Reconstituted Infant Formulae

**DOI:** 10.3389/fmicb.2017.01799

**Published:** 2017-09-21

**Authors:** Nathália Buss da Silva, József Baranyi, Bruno A. M. Carciofi, Mariem Ellouze

**Affiliations:** ^1^Department of Chemical and Food Engineering, Federal University of Santa Catarina Florianópolis, Brazil; ^2^Nestlé Research Center Lausanne, Switzerland; ^3^Institute of Nutrition, University of Debrecen Debrecen, Hungary

**Keywords:** *Bacillus cereus*, predictive microbiology, bias factor, reconstituted infant formulae, Ratkowsky model

## Abstract

Predictive models of the growth of foodborne organisms are commonly based on data generated in laboratory medium. It is a crucial question how to apply the predictions to realistic food scenarios. A simple approach is to assume that the bias factor, i.e., the ratio between the maximum specific growth rate in culture medium and the food in question is constant in the region of interest of the studied environmental variables. In this study, we investigate the validity of this assumption using two well-known link functions, the square-root and the natural logarithm, both having advantageous properties when modeling the variation of the maximum specific growth rate with temperature. The main difference between the two approaches appears in terms of the respective residuals as the temperature decreases to its minimum. The model organism was *Bacillus cereus*. Three strains (B594, B596, and F4810/72) were grown in Reconstituted Infant Formulae, while one of them (F4810/72) was grown also in culture medium to calculate the bias factor. Their growth parameters were estimated using viable count measurements at temperatures ranging from 12 to 25°C. We utilized the fact that, if the bias factor is independent of the temperature, then the minimum growth temperature parameter of the square-root model of Ratkowsky et al. (1982) is the same for culture medium and food. We concluded, supported also by mathematical analysis, that the Ratkowsky model works well but its rearrangement for the natural logarithm of the specific growth rate is more appropriate for practical regression. On the other hand, when analyzing mixed culture data, available in the ComBase database, we observed a trend different from the one generated by pure cultures. This suggests that the identity of the strains dominating the growth of mixed cultures depends on the temperature. Such analysis can increase the accuracy of predictive models, based on culture medium, to food scenarios, bringing significant saving for the food industry.

## Introduction

*Bacillus cereus* is a Gram positive, spore-forming, facultative anaerobic, rod-shaped pathogen (Kotiranta et al., 2000). *Bacillus cereus* strains are ubiquitous in the environment and can be isolated from soil, water and vegetables (Althayer and Sutherland, 2006; El-Arabi and Griffiths, 2013). They are commonly found in raw materials and processed foods, such as rice, milk and dairy products, meat and meat products, pasteurized liquid egg, ready-to-eat vegetables, and spices (Ceuppens et al., 2011). *Bacillus cereus* can be responsible for food poisoning illnesses in two ways: by heat labile, diarrhea-causing enterotoxins produced in the small intestine, and by heat stable cereulide toxin produced in the food before ingestion (Ceuppens et al., 2011), causing emetic syndromes. Emetic strains are, therefore, a concern to the food industry since it is not possible to eliminate the cereulide once performed in the food. Growth and toxin production must be strictly controlled, especially in food targeted to sensitive populations.

*Bacillus cereus* can endure ultrahigh-temperature (UHT) pasteurization, resist spray drying and survive in final products (Mcauley et al., 2014). Moreover, according to a review published by the European Food Safety Agency (EFSA, 2005), *B. cereus* strains are highly variable in terms of their tolerance to high temperatures and their ability to grow. This is mainly dependent on their phylogenetic group (Carlin et al., 2013). Mathematical modeling can be a valuable tool to assess and quantify this variability. It is widely accepted that temperature is the most important external environmental factor affecting microbial response. Among the available predictive models, the model of Ratkowsky et al. (1982) is commonly used to predict the maximum specific growth rate in the suboptimal region of temperature.

However, developing and validating a new model to predict microbial behavior during the manufacturing or the shelf life of a food commodity require extensive experimental work. It is a good practice to use readily available published data and models in the literature or in user-friendly predictive microbiology tools. For example, ComBase (http://www.combase.cc) provides culture-medium-based predictive models for a large collection of micro-organisms including *B. cereus*. To establish a “correction factor” that could be used to predict the behavior of the organism in food from culture-medium based models would be valuable for the food industry. To quantify the similarity between prediction and observation, the accuracy and bias factors, *A*_*f*_ and *B*_*f*_, respectively, of Ross (1996) is commonly used for practical applications. The indicators ln(*A*_*f*_) and ln(*B*_*f*_) are certain average differences between the natural logarithm of the predicted and observed ln(μ) values of the organism in the studied range of environmental conditions, where μ denotes the maximum specific growth rate under a given condition. In the case of *A*_*f*_, the difference is meant as an absolute value, while in the case of *B*_*f*_ it is meant with its sign. Consequently, a bias factor *B*_*f*_ = 1 means that, in a studied region, on average, the model predictions are neither over-estimating nor under-estimating the growth rate compared to the observations. However, this could happen in such a way, too, that the predictions are underestimations in one part of the region while they are overestimations in the other part. It would be desirable that, for a given food matrix, the bias factor is independent of the environmental conditions, primarily of the temperature, at least in the normal physiological growth region of the organism. In this case, culture-medium-based predictions, available from public software tools such as ComBase, could be readily applied to the food in question. Since culture medium broths provide optimal substrate for the organism, the bias factor μ_*food*_*/*μ_*broth*_ should normally be < 1.

The main objectives of this paper are (i) to provide a numerical analysis for the connection between bias and the two most frequently used transformations of the maximum specific growth rate parameter, the square-root and the logarithm functions; and (ii) to test whether the bias factor for *B. cereus* in Reconstituted Infant Formulae (RIF) can be considered constant, at least in a sub-optimal region of the temperature.

## Materials and methods

### Samples preparation

In laminar flow cabinet, infant formulae milk powder was weighted into sterile bottles, warm (~50°C) sterile water was aseptically added and then mixed to dissolve, according to manufacturer's instructions to obtain 50 ml of RIF samples.

### Strains preparation

Three emetic strains of *Bacillus cereus* were used in this study. B594 and B596 isolated from cereals and filed in the Nestlé culture collection and F4810/72 a reference strain from the DSMZ culture collection isolated during an outbreak investigation and also referred to as DSMZ4312 as reported in Carlin et al. (2013). Stock cultures were formed using subcultures of each strain supplemented with glycerol and stored at −80°C until used. For each strain, one tube of frozen stock culture was used to inoculate 10 ml of BHI (Sigma-Aldrich) and stored for 24 h at 30°C. Then 100 μl of this culture was put into another 10 ml of BHI and incubated for 18 h at 30°C. The subculture was then enumerated both on selective (Bacara, BioMérieux) and a non-selective (TSAyE, Sigma-Aldrich) media, diluted to a target level of 10^6^ CFU/ml before applying a thermal stress during 25 s at 72°C. The plates were incubated for 24 h at 30°C. The stressed culture was also enumerated both on the selective and non-selective media to assess the stress intensity. This protocol allowed to mimic the processing conditions that influences the physiological state of naturally contaminating *B. cereus* cells.

### Experimental design

Prior to inoculation, RIF bottles were equilibrated at the targeted temperatures (9, 12, 15, 18, 22, 25°C for F4810/72 strain and 9, 12, 15, 18, and 22°C for B594 and B596 strains). Appropriate dilution of the inoculum was then added to 50 ml of RIF to reach an initial concentration of 2.5 log CFU/ml. Inoculated bottles were sampled for viable counts on Bacara medium at appropriate sampling times to describe the different phases of the growth curves. Three independent replicates were performed for each experiment. For the reference strain F4810/72, additional experiments were performed in BHI following the same protocol to calculate the bias factor. All experiments were performed with pure culture.

### Data analysis

#### Primary and secondary modeling

For each temperature, each curve was fitted by the primary model of Baranyi and Roberts (1994) using the DMFit Excel Add-in, downloadable from http://www.combase.cc/index.php/en/tools. As a second step, the effects of the environment, history and strain were studied and modeled on the maximum specific growth rate (μ), duration of lag phase (λ), and the natural logarithm of the maximum population density (*y*_*max*_).

The maximum population density and the *h*_0_ = μ·λ parameters were taken as constant, as the simplest (zero-order) model, obtained via taking the multiplicative average of their estimates from the primary model.

The model of Ratkowsky et al. (1982),

(1) $$\begin{array}{r} \sqrt{\mu }=a+bT \end{array}$$

in the reparameterized version

(2) $$\begin{array}{r} \sqrt{\mu }=b\left(T-T_{\min}\right) \end{array}$$

was fitted to the square root of the specific growth rates to describe the effect of temperature, where *a* and *b* are constants and *T*_min_ = −*b/a* is a nominal minimum growth temperature, at which the extrapolated maximum specific growth rate would be zero.

We use the above well-established model in its second form, Equation (2), so (though it leads to non-linear regression), the important *T*_min_ parameter and its standard error can directly be obtained. To carry out the non-linear fitting, the method of Levenberg–Marquardt (Press et al., 1986) was programmed in Visual Basic for Applications assigned to MS Excel.

We also tested the Ratkowsky model in a rearranged form, with the natural logarithm link function applied to the observed maximum specific growth rates:

(3) $$\begin{array}{r} \ln\;\mu =\ln\;b^{2}+\ln\;{\left(T-T_{\min}\right)}^{2} \end{array}$$

#### Bias factor

A measure of the deviation between observed and predicted growth, called the “bias factor” was introduced by Ross (1996). As per definition, its natural logarithm, ln(*B*_*f*_) is the average value between the observed and predicted ln(μ) values where μ denotes the maximum specific growth rate of the organism. It is of common sense to expect the conditions (here the temperature), under which the μ-values were generated, randomly distributed in the modeled region, in which case

(4) $$\begin{array}{r} ln\left(B_{f}\right)=E\left[ln\left(\mu_{obs}\right)-ln\left(\mu_{pred}\right)\right] \end{array}$$

where E denotes the “expected value” operator,. Since the μ_*pred*_ predictions produced by commonly used software packages are often based on experiments carried out in culture medium broth, while practical observations (μ_*obs*_) refer to food, the above expectation can be translated to

(5) $$\begin{array}{r} ln\left(B_{f}\right)=E\left[ln\left(\mu_{food}\right)-ln\left(\mu_{broth}\right)\right]. \end{array}$$

In our case, the studied food matrix is RIF, for which a bias factor can be derived via the above formula from the growth rate in broth.

## Results

### Primary and secondary modeling

**Figure 1:**
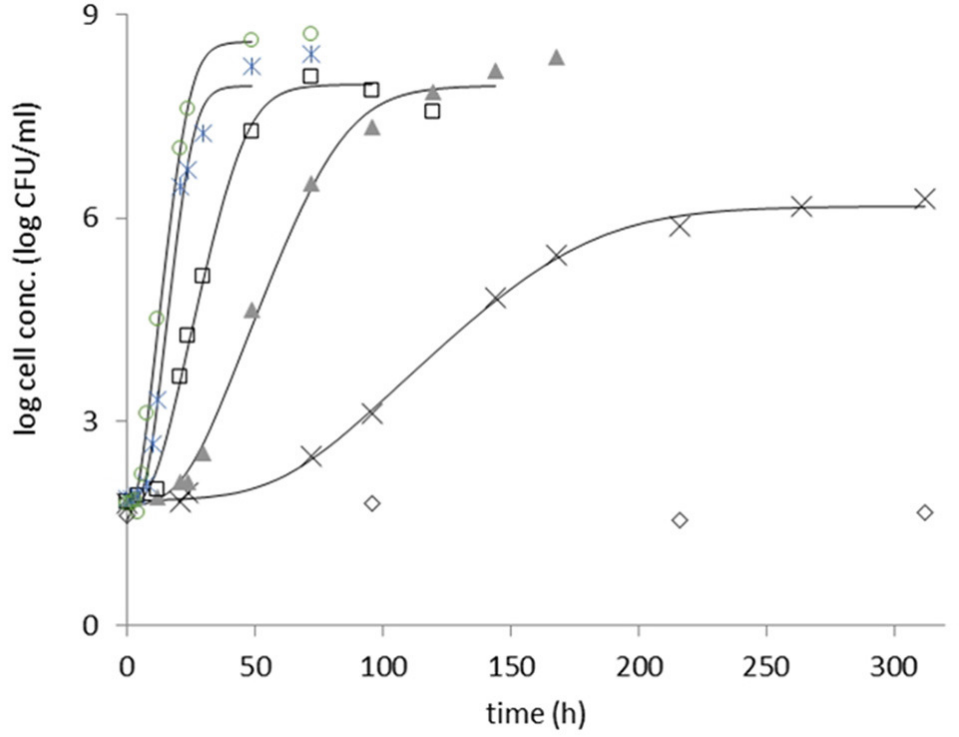
Growth of heat stressed *B. cereus* strain F4810/72, in RFI, at 9°C (diamond), 12°C (cross), 15°C (triangle), 18°C (square), 22°C (star), and 25°C (circle). The sigmoid model of Baranyi and Roberts (1994) was fitted (continuous lines) to the log count curves at each temperature.

Examples for fitting the primary model can be seen in Figure 1.

**Figure 2:**
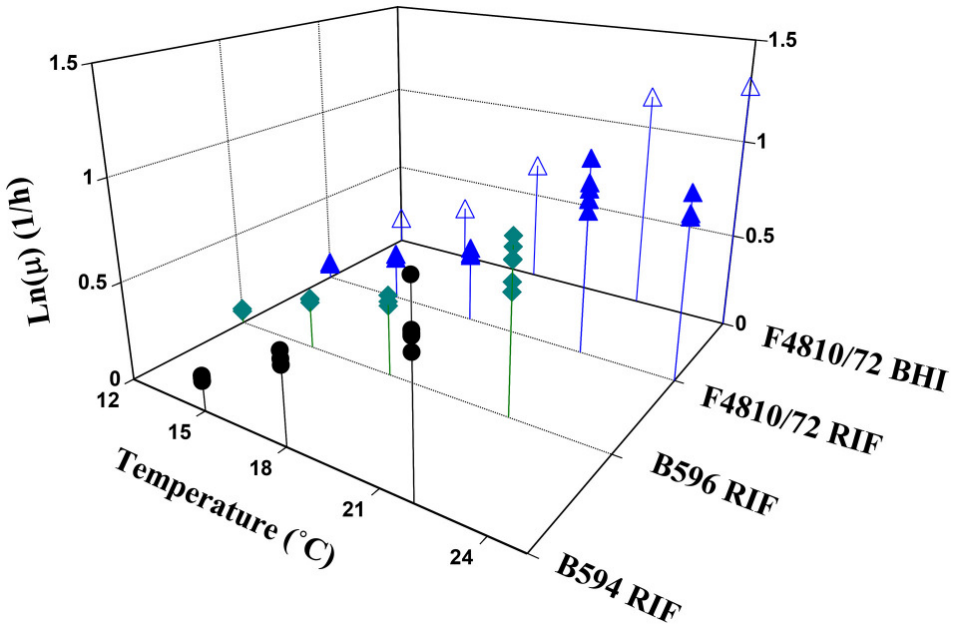
Maximum specific growth rate estimates as a function of temperature for all analyzed strains.

The parameter estimates from the primary modeling are given in the Supplementary Information. Figure 2 shows all specific rate estimates for the three studied strains at the different temperatures in broth and in RIF.

The estimates for the *b* and *T*_min_ parameters, when the Ratkowsky model was fitted to the maximum specific growth rates, are shown in Table 1. The *T*_min_-estimate for the strain B594 was significantly higher than the respective estimates for the other two strains.

**Table 1:** Estimated parameters and their standard errors for the square root model in RIF (3 strains) and BHI (one strain).

**Strain and medium**	***T*_min_ (°C)**	***b* (h^−1/2^/°C)**
B594, RIF	8.43 ± 0.86	0.0643 ± 0.005
B596, RIF	6.52 ± 0.72	0.0555 ± 0.003
F4810/72, RIF	5.40 ± 0.88	0.0510 ± 0.003
F4810/72, BHI	5.13 ± 1.12	0.0601 ± 0.005

**Figure 3:**
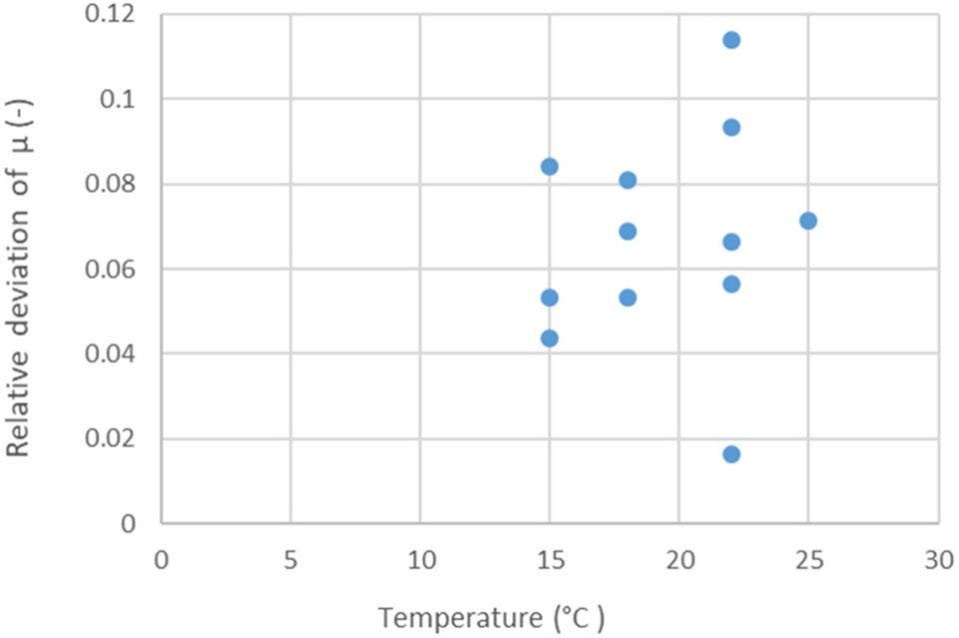
Relative deviations (standard deviation divided by the mean) of replicate specific growth rate estimates, in RIF, at the studied temperatures. There is no correlation (*p* = 0.65) between the relative errors and the temperature.

The Ratkowsky model claims two major benefits: the linear model structure for the “$\sqrt{\mu }$ vs. temperature” relationship, and the constant variance of the measured $\sqrt{\mu }$ values, independently of the temperature. In our case, because of biological replicates (three), it was possible to study the relative deviations of the specific growth rates (standard deviation divided by the mean) within the replicate curve-triplets. These showed no correlation with the temperature (*p* = 0.65), see Figure 3.

This suggests that the natural logarithm could also be a suitable link function for the maximum specific growth rate when modeling its dependence on the temperature. This comes from the relationships:

(6) $$\begin{array}{r} \frac{\mu_{obs}-\;\mu }{\mu }=\varepsilon \end{array}$$

(7) $$\begin{array}{r} \mu_{obs}\;=\;\mu \left(1+\varepsilon \right) \end{array}$$

(8) $$\begin{array}{r} \ln\;\left(\mu_{obs}\right)=\ln\;\left(\mu \right)+\ln\;\left(1+\varepsilon \right)\approx \ln\;\left(\mu \right)+\varepsilon \end{array}$$

where the approximation is accurate at least for one digit if the relative error ε is less than 0.3. For ε-values over 0.3, the approximation in Equation (8) would have worse than one digit accuracy. From this, it also follows that, since our average relative errors are less than 10%, the standard error of fit of the secondary model for ln(μ) will be ca 0.1 (or possibly higher, if the secondary model describes the “μ vs. *T*” relationship inaccurately).

It can be readily seen that if the ε random error in the Equations (6–8) is constant, independently of the temperature, then the same cannot hold for the square-root model and vice versa. Revisiting Equation (7), one can obtain, by first order approximation:

(9) $$\begin{array}{r} \sqrt{\mu_{obs}}=\sqrt{\mu }\cdot \sqrt{\left(1+\varepsilon \right)}\approx \sqrt{\mu }+\frac{\sqrt{\mu }}{2}\cdot \varepsilon \end{array}$$

This means that, if the natural logarithm transformation makes the deviation of the observed specific rates constant, then the deviation generated by the square-root function should tend to be smaller with temperature decreasing to *T*_min_. That is, the absolute residuals should show a decreasing trend with the temperature (and, therefore, with the μ-values) - as indeed we will see it later. On the other hand, if the square-root was the correct transformation to stabilize the variance still the natural logarithm of the μ-values is regressed in the secondary model, then the residuals should show increasing trend as the temperature decreases to *T*_min_:

(10) $$\begin{array}{r} \sqrt{\mu_{obs}}\;=\;\sqrt{\mu }+\delta \end{array}$$

(11) $$\begin{array}{r} \ln\;\mu_{obs}\approx \ln\;\mu +\text{ln}{\left(1+\frac{\delta }{\sqrt{\mu }}\right)}^{2} \end{array}$$

### Bias factor

The maximum specific growth rate of the strain F4810/72 was measured in both RIF and BHI, providing a good opportunity to investigate the bias factor. Notice that, if this is independent of the temperature, then the secondary model for ln(μ) for the two media (Equation 3) differ only by an additive constant (Equation 5). This is equivalent to the assumption that the *T*_min_ parameter is the same for the BHI and RIF. Our investigations showed that the *T*_min_ of this same strain in BHI was 5.13 ± 1.12 which is not significantly different (*p* = 0.35) from the *T*_min_-value in RIF 5.40 ± 0.88 (Table 1).

Then we use the formulae

(12) $$\begin{array}{r} \sqrt{\mu_{broth}}=b_{broth}\left(T-T_{\min}\right) \end{array}$$

(13) $$\begin{array}{r} \sqrt{\mu_{food}}=b_{food}\left(T-T_{\min}\right) \end{array}$$

from which the ratio *B*_*f*_ = (*b*_*food*_/*b*_*broth*_)^2^ is constant, so the secondary models for ln(μ), for broth and food, should be parallel and differ from each other by the

(14) $$\begin{array}{r} ln\left(B_{f}\right)=2\text{ln}\left(b_{Food}/b_{Broth}\right) \end{array}$$

constant additive term. The opposite direction of this conclusion can be proven similarly, therefore, the assumption that *T*_min_ is the same for culture medium and food is equivalent to the one that the ratio between maximum specific growth rates in culture medium broth and food is constant, independently of the temperature. In our situation, we showed, by *F*-test, that the strain F4810/72 has the same *T*_min_ = 5.26 value for BHI and RIF (*p* = 0.35). Therefore, their model can be written as

$$\begin{array}{l} \sqrt{\mu_{RIF}}=0.050\left(T-5.26\right)\\ \sqrt{\mu_{BHI}}=0.061\left(T-5.26\right) \end{array}$$

Substituting the coefficients above in Equation (14), it can be calculated that, for this strain, the ratio between the maximum specific growth rates in RIF and culture medium broth is *B*_*f*_ = (0.050**/**0.061)^2^ = 0.67. That is, this strain grows at one third slower in RIF compared to BHI.

**Figure 4:**
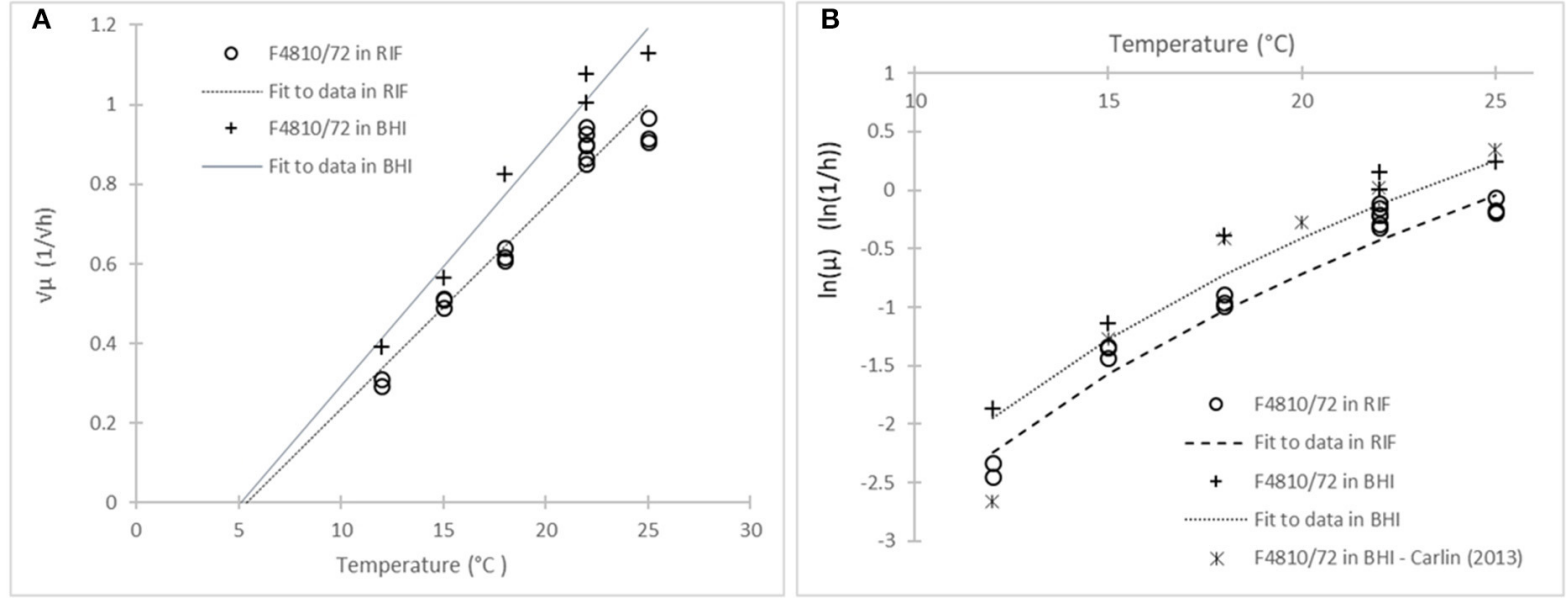
Model of Ratkowsky et al. (1982) with two different link functions: **(A)** Square-root; and **(B)** natural logarithm. The model describes the effect of temperature on the maximum specific growth rate of the strain F4810/72 in RIF (circles) and in BHI (crosses). The **(B)** plot also shows data from Carlin et al. (2013) (stars) for the same strain in broth.

Figure 4 demonstrates well the equivalence between the two assumptions: common *T*_min_-value for the square-root and parallel models for the logarithm link functions. The model (3) fitted to the ln(μ) values of the strain F4810/72 in BHI and RIF are parallel because they have similar *T*_min_ estimates. As follows from Equation (9), the deviation from the parallel behavior would be apparent at low temperatures only.

**Figure 5:**
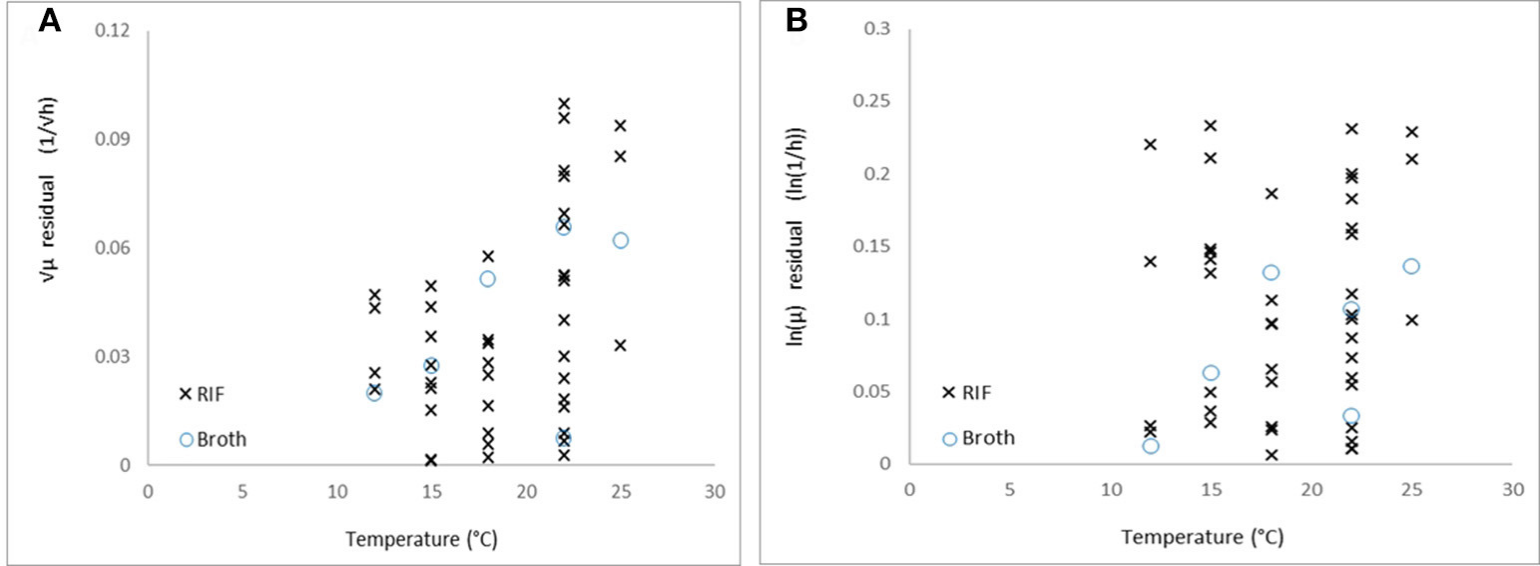
Absolute residuals vs. temperature for **(A)** the square-root and **(B)** natural logarithm link functions.

The shown equivalence is independent of the question whether the square-root or the logarithm transformation stabilizes the variance of the μ-values. According to the Equations (7) and (9), both cannot be valid at the same time. Comparing the absolute residuals for both the square-root and logarithm link function, on all the data, the Figure 5 emerges.

The residuals with the square-root link function show a decreasing trend as the temperature decreases to *T*_min_ (*p* = 0.004), while in the case of logarithm link function, it does not show correlation with the temperature (*p* = 0.37). Therefore, based on our data, while the Ratkowsky model accurately describes how the maximum specific growth rate depends on temperature, the logarithm link function is more suitable to be applied to the observed maximum specific growth rates when regressing them against temperature. The difficulty is that this difference between the two link functions can be detected at low temperatures only, where it is not easy to keep the environment constant for the required long time to reach the stationary phase, therefore, the environmental effects (e.g., pH decrease in the medium) rather than biological ones (linked to strain variability for example) can dominate the variability of the observed maximum specific growth rates.

## Discussion

The paper of Carlin et al. (2013) gives an opportunity to compare the kinetic parameters of the reference strain F4810/72 in broth as shown in Figure 4. Fitting the square-root model to the 12–25°C data for the same strain in that paper, the estimated parameters were not different at 5% significance level (*p* = 0.12).

**Figure 6:**
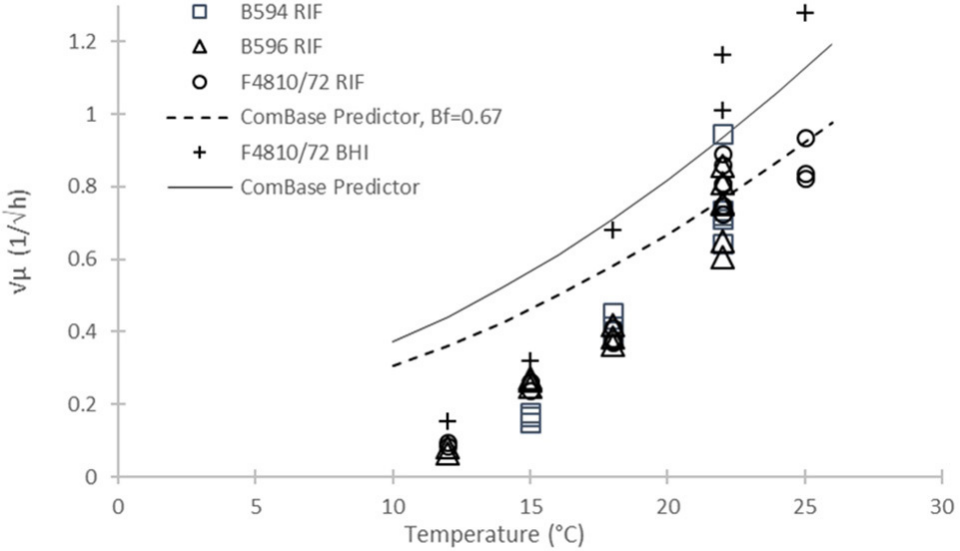
Square root of maximum specific growth rates of *B. cereus* vs. temperature from different sources: Our B594, B596, and F4810/72 strains in RIF (open diamond, circle and triangle, resp.); Our data for F4810/72 in broth (plus sign); and ComBase Predictor results (continuous line). The dashed line below the ComBase Predictor curve is the prediction obtained by using the *B*_*f*_ = 0.67 bias factor.

In the same way, we can validate our rate estimates by the ComBase Predictor available from http://www.combase.cc. In Figure 6, the square root values of our specific growth rates can be compared with results from ComBase Predictor, in broth and milk, assuming a bias factor of *B*_*f*_ = 0.67 for the food scenario.

The validation plot in Figure 6 is a convincing proof of the diversity of the kinetic behavior of *B. cereus* strains. The ComBase Predictor is based on growth curves generated by a cocktail of six strains (Sutherland et al., 1996). A plausible explanation for the seemingly unexpected non-linear behavior of the $\sqrt{\mu }$ predictions is that different strains were the dominant ones at different temperatures, while the same parameter of a pure culture show a consistent linear pattern with temperature.

For quantitative validation, we made an extensive use of the Bias and Accuracy factors of Ross (1996). We point out here that while acknowledging the useful applicability of these indicators, their definition needs some refinement, in agreement with Baranyi et al. (1999). When the average of the ln(μ_*food*_) - ln(μ_*pred*_) values is taken, it is implicitly assumed that the probability distribution of this difference is independent of the temperature and possibly other environmental factors (Gill and Phillips, 1985; Buchanan and Bagi, 1997; Neumeyer et al., 1997; Mellefont et al., 2003). The constant bias-factor is a reasonable assumption in case of the temperature, with the rationale that all affecting biochemical reactions speed up or slow dow when temperature changes. It is less obvious with other environmental factors, like pH or water activity; nonetheless the assumption provides a convenient approximation that is easy to build in predictive packages.

The assumption of the temperature-independent bias factor is equivalent to the existence of a minimum growth temperature that is the same for the model and for the food matrix on which the model is tested. Indeed, this latter condition has been assumed by quite a few authors (Miles et al., 1997; Carlin et al., 2013; Aryani et al., 2015, 2016), and was observed in our situation, too, when comparing the temperature-dependent maximum specific growth rates in RIF and culture medium. The ComBase database (http://www.combase.cc) also provides a good opportunity to check how much the temperature-independence of the Bias factor is tenable.

In conclusion, we agree with many authors (Bernaerts et al., 2000; Ross et al., 2003; Powell et al., 2015; Den Besten et al., 2017) that, at sub-optimal temperatures, the Ratkowsky model is a good representation of the effect of temperature on the maximum specific growth rate measured in a pure culture, in both laboratory medium and food. However, the constant variance assumption does not necessarily hold at low temperatures. Besides, we established that the minimum growth temperature can be taken as the same *T*_min_ value for culture medium and food, therefore, the bias factor is, indeed, independent of the temperature. In mixed cultures, however none of the above holds, and more complex developments (data and mathematical considerations) are needed for an accurate model; see Baranyi et al. (2017), which is, in a sense, a continuation of this paper.

## Author contributions

NBdS: experiments and writing up. JB Experimental design, data analysis and writing up. BAMC Consultation. ME conception, experimental design and writing up.

### Conflict of interest statement

The authors declare that the research was conducted in the absence of any commercial or financial relationships that could be construed as a potential conflict of interest.
