# Supplementary material for: From Culture-Medium-Based Models to Applications to Food: Predicting the Growth of B. cereus in Reconstituted Infant Formulae
Source: Front Microbiol. 2017 Sep 21;8:1799. doi: 10.3389/fmicb.2017.01799 (PMC5613307; doi:10.3389/fmicb.2017.01799)
Supplement: Supplementary file 1 [file DataSheet1.docx]

# **Supplementary Information**

# Kinetic parameters obtained by fitting experimental data.

nm = not measured; nd = not detected (less than 10 cells/100 ml). Bold italics: omitted from analysis as outliers.

| Strain | Experiment* | Culture medium | Average T(°C) | Number of data points | Inoculum log (cell /ml) | Rate (log (cells/ml)/h) | se** (rate) (log (cell/ml)/h) | Lag time (h) | se**(lag) (h) | h0 | se**(h0) | Max. pop. dens. (log (cell /ml)) | se**(Max.pop.d) (log (cell /ml)) |
| --- | --- | --- | --- | --- | --- | --- | --- | --- | --- | --- | --- | --- | --- |
| B594 | 15 – A | RIF | 15.07 | 16 | 2.44 | 0.0769 | 0.0033 | 26.23 | 1.67 | 4.66 | 8.93 | 7.33 | 0.0421 |
| B594 | 15 – B | RIF | 15.00 | 13 | 1.90 | 0.0650 | 0.0047 | 13.60 | 4.27 | 2.04 | 19.64 | 7.94 | 0.1240 |
| B594 | 15 – C | RIF | 15.04 | 11 | 2.02 | 0.0720 | 0.0069 | 19.10 | 4.75 | 2.87 | 22.98 | 7.28 | 0.1114 |
| B594 | 18 – A | RIF | 18.15 | 12 | 2.38 | 0.1800 | 0.0175 | 12.40 | 1.79 | 5.27 | 11.42 | 8.21 | 0.0899 |
| B594 | 18 – B | RIF | 18.06 | 10 | 1.89 | 0.1676 | 0.0241 | 8.89 | 3.27 | 3.51 | 18.16 | 8.23 | 0.1762 |
| B594 | 18 – C | RIF | 18.09 | 9 | 2.03 | 0.1969 | 0.0229 | 10.90 | 2.30 | 4.62 | 15.60 | 8.33 | 0.1180 |
| B594 | 22 – A | RIF | 22.15 | 12 | 2.36 | 0.3128 | 0.0346 | 7.33 | 1.22 | 5.95 | 7.04 | 8.24 | 0.1503 |
| B594 | 22 – B | RIF | 21.96 | 11 | 1.98 | 0.3187 | 0.0232 | 6.25 | 0.77 | 4.58 | 4.82 | 7.98 | 0.1321 |
| B594 | 22 – C | RIF | 21.99 | 11 | 1.99 | 0.3084 | 0.0250 | 6.96 | 0.97 | 4.61 | 5.34 | 8.12 | 0.1662 |
| B594 | 22UH - B | RIF | 21.96 | 8 | 2.69 | 0.2802 | 0.0355 | 1.83 | 1.52 | 1.18 | 10.68 | 8.12 | 0.1271 |
| B594 | 22UH - C | RIF | 21.99 | 11 | 2.89 | 0.4110 | 0.0586 | 3.46 | 1.06 | 3.12 | 8.31 | 8.11 | 0.1020 |
|  |  |  |  |  |  |  |  |  |  |  |  |  |  |
| B596 | 12 – A | RIF | 11.95 | 11 | 2.04 | 0.0288 | 0.0022 | 96.40 | 8.56 | 6.39 | 36.29 | 7.12 | 0.1022 |
| B596 | 12 – B | RIF | 12.10 | 8 | 1.84 | 0.0349 | 0.0096 | 103.00 | 18.26 | 9.33 | 90.30 | - | 1.3750 |
| ***B596*** | ***12 – C*** | ***RIF*** | ***12.09*** | ***11*** | ***1.72*** | ***0.0155*** | ***0.0015*** | ***21.40*** | ***16.07*** | ***0.76*** | ***76.13*** | ***5.43*** | ***0.0976*** |
| B596 | 15 – A | RIF | 15.04 | 12 | 1.96 | 0.1081 | 0.0053 | 24.20 | 1.57 | 5.96 | 9.03 | 7.55 | 0.0551 |
| B596 | 15 – B | RIF | 15.04 | 12 | 1.76 | 0.1151 | 0.0063 | 26.20 | 1.68 | 6.99 | 10.05 | 7.56 | 0.0603 |
| B596 | 15 – C | RIF | 15.05 | 12 | 1.76 | 0.1177 | 0.0066 | 25.70 | 1.70 | 6.96 | 10.05 | 7.59 | 0.0620 |
| B596 | 18 – A | RIF | 18.12 | 10 | 1.91 | 0.1584 | 0.0123 | 9.39 | 1.72 | 3.46 | 10.53 | 7.80 | 0.0863 |
| B596 | 18 – B | RIF | 18.12 | 10 | 1.66 | 0.1676 | 0.0119 | 11.60 | 1.57 | 4.50 | 9.15 | 8.00 | 0.0941 |
| B596 | 18 – C | RIF | 18.13 | 10 | 1.69 | 0.1816 | 0.0111 | 12.10 | 1.24 | 5.13 | 8.25 | 8.10 | 0.0755 |
| B596 | 22 – A | RIF | 22.97 | 11 | 2.08 | 0.3263 | 0.0389 | 8.27 | 1.21 | 6.05 | 6.74 | 7.43 | 0.1981 |
| B596 | 22 – B | RIF | 21.94 | 11 | 1.72 | 0.3518 | 0.0063 | 9.55 | 0.20 | 7.67 | 5.36 | 7.52 | 0.0326 |
| B596 | 22 – C | RIF | 22.14 | 11 | 1.77 | 0.3728 | 0.0188 | 9.22 | 0.50 | 7.89 | 5.64 | 7.79 | 0.0863 |
| B596 | 22UH - A | RIF | 22.97 | 10 | 2.19 | 0.2830 | 0.0188 | 1.77 | 0.68 | 1.16 | 4.84 | 7.70 | 0.0687 |
| B596 | 22UH - B | RIF | 21.94 | 11 | 2.18 | 0.2631 | 0.0315 | 1.35 | 1.43 | 0.81 | 7.86 | 7.70 | 0.1463 |
| B596 | 22UH - C | RIF | 22.14 | 11 | 2.19 | 0.3279 | 0.0277 | 3.18 | 0.75 | 2.39 | 6.14 | 7.84 | 0.0798 |
|  |  |  |  |  |  |  |  |  |  |  |  |  |  |
| F4810/72 | 12 – A | RIF | 12.06 | 10 | 1.80 | 0.0375 | 0.0024 | 57.90 | 5.00 | 5.21 | 22.28 | 6.28 | 0.0663 |
| F4810/72 | 12 – B | RIF | 11.93 | 10 | 1.80 | 0.0421 | 0.0025 | 69.30 | 4.98 | 6.47 | 23.35 | 6.97 | 0.0807 |
| ***F4810/72*** | ***12 – C*** | ***RIF*** | ***11.93*** | ***9*** | ***1.51*** | ***0.0252*** | ***0.0026*** | ***37.50*** | ***11.16*** | ***2.43*** | ***52.35*** | ***5.66*** | ***0.1186*** |
| F4810/72 | 15 – A | RIF | 15.09 | 11 | 1.82 | 0.1039 | 0.0107 | 23.30 | 4.02 | 5.43 | 20.42 | 8.12 | 0.1422 |
| F4810/72 | 15 – B | RIF | 15.09 | 11 | 1.67 | 0.1144 | 0.0107 | 23.90 | 3.32 | 6.64 | 17.82 | 8.13 | 0.1185 |
| F4810/72 | 15 – C | RIF | 15.06 | 10 | 1.49 | 0.1138 | 0.0088 | 25.20 | 2.75 | 6.34 | 14.79 | 7.50 | 0.0983 |
| F4810/72 | 18 – A | RIF | 18.17 | 9 | 1.81 | 0.1786 | 0.0219 | 11.40 | 2.42 | 4.23 | 12.77 | 7.82 | 0.1474 |
| F4810/72 | 18 – B | RIF | 18.17 | 9 | 1.74 | 0.1611 | 0.0210 | 9.80 | 3.31 | 3.21 | 15.79 | 8.58 | 0.2154 |
| F4810/72 | 18 – C | RIF | 18.11 | 8 | 1.25 | 0.1662 | 0.0245 | 8.58 | 3.74 | 3.30 | 20.59 | 8.12 | 0.2097 |
| F4810/72 | 22 – A | RIF | 22.02 | 10 | 1.87 | 0.3512 | 0.0406 | 8.07 | 1.43 | 6.32 | 7.42 | 8.07 | 0.2418 |
| F4810/72 | 22 – B | RIF | 22.00 | 10 | 1.68 | 0.3871 | 0.0343 | 7.78 | 1.06 | 7.07 | 6.25 | 8.39 | 0.1926 |
| F4810/72 | 22 – C | RIF | 21.98 | 8 | 1.40 | 0.3521 | 0.0116 | 6.64 | 0.37 | 6.61 | 5.02 | 7.99 | 0.0678 |
| F4810/72 | 25 – A | RIF | 24.90 | 10 | 1.73 | 0.3575 | 0.0310 | 4.87 | 1.17 | 3.34 | 6.03 | 8.65 | 0.2179 |
| F4810/72 | 25 – B | RIF | 24.90 | 11 | 1.76 | 0.4070 | 0.0133 | 6.12 | 0.32 | 5.56 | 3.90 | 8.40 | 0.0630 |
| F4810/72 | 25 – C | RIF | 24.97 | 10 | 1.35 | 0.3644 | 0.0461 | 4.19 | 1.16 | 4.08 | 6.50 | 8.25 | 0.1920 |
| F4810/72 | 22UH - A | RIF | 22.02 | 12 | 1.20 | 0.3259 | 0.0246 | 1.62 | 1.14 | 0.92 | 5.26 | 8.48 | 0.1740 |
| F4810/72 | 22UH - B | RIF | 22.00 | 10 | 1.53 | 0.3144 | 0.0280 | 0.00 | 1.29 | 0.00 | 6.60 | 8.46 | 0.1552 |
| F4810/72 | 22UH - C | RIF | 21.98 | 10 | 2.64 | 0.3739 | 0.0740 | 0.00 | 1.58 | 2.47 | 11.29 | 8.11 | 0.1309 |
| F4810/72 | 12 – C | BHI | 11.93 | 8 | 1.60 | 0.0670 | 0.0220 | 50.50 | 13.74 | 7.70 | 81.95 | 5.99 | 0.2134 |
| F4810/72 | 15 – C | BHI | 15.06 | 9 | 1.69 | 0.1388 | 0.0111 | 24.40 | 2.15 | 7.80 | 12.85 | 7.62 | 0.0926 |
| F4810/72 | 18 – C | BHI | 18.11 | 7 | 1.57 | 0.2952 | 0.2713 | 13.20 | 10.55 | 8.79 |  | 6.92 | 0.2773 |
| F4810/72 | 22 – C | BHI | 21.98 | 10 | 1.42 | 0.5055 | 0.1252 | 6.76 | 1.18 | 8.57 | 12.82 | 7.70 | 0.1279 |
| F4810/72 | 25 – C | BHI | 24.97 | 10 | 1.57 | 0.4391 | 0.0685 | 5.66 | 0.99 | 4.24 | 7.81 | 7.77 | 0.1101 |
| F4810/72 | 22UH - C | BHI | 21.98 | 10 | 2.90 | 0.5556 | 0.0592 | 4.16 | 0.59 | 7.22 | 9.11 | 7.46 | 0.0743 |

* Experiment: temperature (in ˚C) followed by replicate code (A, B or C), UH = unheated cells; **se =standard error.
